# Translated Circular RNAs in Glioblastoma: Emerging Regulators of Tumor Progression, Molecular Heterogeneity and Therapeutic Resistance

**DOI:** 10.3390/ijms27198743

**Published:** 2026-09-30

**Authors:** Robert N. Nishimura, Joseph F. Gera

**Affiliations:** 1 Departments of Neurology, David Geffen School of Medicine at UCLA, Los Angeles, CA 90095, USA; 2 Department of Research & Development, Greater Los Angeles Veterans Affairs Healthcare System, 16111 Plummer Street (151), Building 1, Room C111A, Los Angeles, CA 91343, USA; 3 Departments of Medicine, David Geffen School of Medicine at UCLA, Los Angeles, CA 90095, USA; 4 Jonsson Comprehensive Cancer Center, University of California-Los Angeles, Los Angeles, CA 90024, USA; 5 Molecular Biology Institute, University of California-Los Angeles, Los Angeles, CA 90095, USA

**Keywords:** glioblastoma, circular RNA translation, non-canonical translation, circRNA-encoded proteins, therapeutic resistance, biomarker, precision oncology

## Abstract

Glioblastoma (GBM) is the most common and highly malignant primary brain tumor in adults. Even with advances in surgery, radiotherapy, and systemic therapies, patient outcomes remain poor due to extensive intratumoral heterogeneity and the development of therapeutic resistance. Circular RNAs (circRNAs) are a class of covalently closed RNA molecules that are highly abundant in the central nervous system and frequently dysregulated in cancer. While originally considered non-coding transcripts, current evidence demonstrates that a subset of circRNAs contain functional open reading frames and can undergo cap-independent translation to generate biologically active proteins and peptides. Recent studies have identified a growing number of translated circRNAs in GBM encoding proteins implicated in diverse processes such as receptor tyrosine kinase signaling, DNA repair, metabolism, stemness, invasion, and therapeutic resistance. These findings expand the coding potential of the GBM transcriptome and highlight an additional layer of post-transcriptional regulation. In this review, we summarize our current understanding of circRNA translation, discuss the biological functions of translated circRNAs in GBM, and examine their potential roles in tumor progression, therapeutic resistance, and clinical management. Additionally, we consider the opportunities and challenges associated with targeting translated circRNAs and their encoded proteins for the treatment of GBM.

## 1. Introduction

Glioblastoma (GBM) is a highly aggressive primary malignant brain tumor in adults and carries a poor prognosis despite maximal safe resection, radiotherapy, and temozolomide (TMZ) [1,2,3,4]. Nearly all tumors recur, reflecting extensive intratumoral heterogeneity and the ability of GBM cells to adapt to microenvironmental and therapeutic stress [2,3,4]. These adaptive responses include changes in signaling, metabolism, cell state, DNA-damage responses, and protein synthesis. Recent transcriptomic studies have shown that this complexity extends beyond conventional messenger RNAs to circular RNAs (circRNAs), endogenous transcripts generated by back-splicing in which a downstream splice donor is covalently joined to an upstream splice acceptor [5,6,7]. Their closed structure lacks free 5′ and 3′ ends, conferring resistance to exonuclease degradation and increased stability relative to many linear RNAs [5,6,7].

CircRNAs are particularly abundant in the central nervous system and display developmental, cell-type-specific, and activity-dependent regulation [8,9,10]. In glioma, altered circRNA expression has been linked to proliferation, invasion, stemness, angiogenesis, metabolism, and therapeutic resistance [10,11,12,13]. Although circRNAs were initially studied primarily as non-coding regulators of microRNAs, RNA-binding proteins, and transcription, a subset contains functional open reading frames and can be translated through cap-independent mechanisms [14,15,16,17]. IRES-dependent initiation and m^6^A-mediated ribosome recruitment are the best established mechanisms, while some circular templates can also support rolling-circle translation [14,15,16,17]. Thus, circRNAs can contribute to gene regulation at both the RNA and protein levels.

For clarity, throughout this review we use “translated circRNA” to refer to a circRNA for which translation is experimentally supported or specifically under consideration, and “circRNA-encoded protein” or “circRNA-encoded peptide” for the resulting translation product. We avoid using “protein-coding circRNA” as the default designation because many circRNAs may have dual functions, acting both as RNA regulatory molecules and as templates for translation.

Several translated circRNAs have been reported and functionally characterized in GBM. Tumor-suppressive examples include circFBXW7, circSHPRH, circAKT3, circLINC-PINT, circNTRK2, and circHEATR5B, whereas circHGF and circEGFR generate products with oncogenic activities [18,19,20,21,22,23,24,25,26]. Collectively, the reported proteins regulate pathways ranging from c-MET and EGFR signaling to c-Myc stability, AKT activation, transcriptional elongation, DNA repair, and glycolysis. These observations expand the coding potential of the GBM transcriptome and suggest that circRNA translation may contribute to molecular heterogeneity and stress adaptation, particularly when canonical cap-dependent translation is suppressed [13].

Several previous reviews have discussed the emerging capacity of circRNAs to encode proteins in glioma and GBM [27,28]. The present review extends these earlier syntheses by integrating the mechanisms that support circRNA translation with the experimental approaches used to establish translation and protein function and by critically examining the evidence for individual translated circRNAs characterized in GBM. Particular emphasis is placed on how circRNA-encoded proteins converge on signaling, metabolic, and stress-response pathways relevant to tumor progression and therapeutic response, as well as on the methodological limitations that currently constrain interpretation and clinical translation. Finally, we consider the potential clinical implications of translated circRNAs, including their development as biomarkers and therapeutic targets, and highlight key challenges and opportunities for future investigation in neuro-oncology.

## 2. Circular RNAs in GBM

### 2.1. CircRNA Biogenesis and Classification

CircRNAs are generated by back-splicing, producing a covalently closed RNA molecule lacking free termini (Figure 1A) [6,29,30]. Circularization can be promoted by intronic complementary sequences, particularly inverted Alu elements, that bring splice sites into close proximity. RNA-binding proteins, including QKI, MBL, FUS, and NF90/NF110, can further promote or inhibit back-splicing, providing regulated control over circRNA production [6,31,32,33,34]. CircRNAs are generally classified as exonic circRNAs, circular intronic RNAs, or exon-intron circRNAs (Figure 1B) [6,35,36]. Exonic circRNAs are predominantly cytoplasmic and represent the major class relevant to translation, whereas intron-containing forms are frequently nuclear and can influence transcription. The circular architecture also increases resistance to exonuclease-mediated decay and can prolong intracellular persistence. These properties may be especially relevant in GBM, where cells are exposed to hypoxia, nutrient limitation, oxidative stress, and therapy-induced changes in RNA metabolism [10,12]. However, direct evidence that the increased stability of circRNAs itself drives stress-adaptive phenotypes in GBM has not yet been established.

### 2.2. CircRNA Expression in the Normal Brain

The brain contains among the highest levels of circRNAs in mammalian tissues (Figure 1C) [8,9]. Many neuronal circRNAs are developmentally regulated, evolutionarily conserved, and enriched in mature neurons and synaptic compartments, with abundance often increasing during differentiation and aging [8,9,37]. Their accumulation likely reflects both regulated biogenesis and unusual stability. Physiological circRNA functions include microRNA sequestration, interaction with RNA-binding proteins, modulation of transcription, and, for a subset, protein synthesis [10,17]. Because neural cells naturally express a large and diverse circRNA repertoire, brain tumors arise in a tissue context already permissive for extensive circular RNA biology. This makes it important to distinguish cancer-specific changes from the elevated baseline abundance of circRNAs in normal brain [38,39].

### 2.3. Dysregulated CircRNAs in GBM

RNA-sequencing studies have demonstrated widespread circRNA dysregulation in GBM (Figure 1D) [11,40,41,42,43,44]. Both oncogenic and tumor-suppressive circRNAs have been reported, and expression patterns can vary with tumor grade, molecular state, and clinical outcome [11,12,41]. Dysregulated circRNAs can influence signaling, cell-cycle progression, invasion, stemness, and therapeutic response, and some are readily detected in extracellular vesicles and biological fluids, increasing interest in their biomarker potential [45]. Importantly, coding and non-coding activities are not mutually exclusive. A circular transcript may alter cellular behavior through RNA-mediated interactions while also serving as a template for a biologically active protein, complicating functional assignment but also expanding the regulatory potential of the transcript.

An important limitation of the existing literature is that several foundational studies predate the WHO CNS5 classification and analyzed cohorts described broadly as glioma or by histologic grade without IDH stratification. Under WHO CNS5, glioblastoma denotes an IDH-wildtype adult-type diffuse astrocytic glioma, whereas IDH-mutant grade 4 tumors are classified as astrocytoma, IDH-mutant, CNS WHO grade 4 [46]. Accordingly, throughout this review we use glioma or diffuse glioma when the primary study did not establish an IDH-wildtype glioblastoma diagnosis and reserve GBM for studies in which that designation is supported. This distinction is important because IDH-mutant and IDH-wildtype diffuse gliomas differ in molecular biology and clinical behavior, and circRNA expression or biomarker associations derived from mixed or historically classified cohorts may not generalize directly to contemporary IDH-wildtype GBM. Future studies should therefore report molecular classification and evaluate translated circRNAs within defined WHO CNS5 entities.

### 2.4. Established Non-Coding Functions of CircRNAs in GBM

The best-characterized non-coding circRNA function is microRNA sequestration, in which circular transcripts act as competing endogenous RNAs that reduce the availability of specific microRNAs for target repression [7,10]. CircRNAs can also bind RNA-binding proteins, alter protein localization or stability, serve as molecular scaffolds, and influence transcription (Figure 1E) [10,17,47]. In GBM, examples such as circNT5E, circHIPK3, and circPOSTN have been linked to oncogenic signaling, invasion, and stem-like phenotypes [48,49,50]. These RNA-mediated functions remain an important part of circRNA biology and provide context for the protein-coding field. The subsequent demonstration that some circRNAs encode functional proteins, however, established a mechanistically distinct layer of regulation and shifted attention from circRNA abundance alone to the question of which circular transcripts are actually translated in tumor cells.

## 3. Evidence for Circular RNA Translation

### 3.1. Discovery of Protein-Encoding Circular RNAs

CircRNAs were long considered non-coding because they lack the 5′ cap and poly(A) tail used by most eukaryotic mRNAs. Evidence for translation emerged when specific circRNAs were found in ribosome- and polysome-associated fractions and when proteins or peptides uniquely attributable to circular templates were subsequently detected (Table 1) [14,15,16]. CircZNF609 provided an early endogenous example, and genome-wide studies later identified numerous candidate translated circRNAs [14,15,16]. Particularly persuasive evidence comes from translation across the back-splice junction (BSJ), which can generate peptide sequences absent from the corresponding linear transcript. These findings established circRNAs as a potential source of previously unrecognized protein diversity and raised the possibility that their translation could be favored during cellular stress.

### 3.2. Mechanisms of CircRNA Translation

#### 3.2.1. IRES-Dependent Translation

IRES-dependent initiation is one of the principal mechanisms used by translated circRNAs. IRES elements recruit the translation machinery internally, bypassing the requirement for recognition of a 5′ cap [14,51,52]. Their activity is often influenced by IRES *trans*-acting factors (ITAFs), including hnRNP A1, PTBP1, La/SSB, PCBP2, and IGF2BP1, which can remodel RNA structure or facilitate recruitment of the 40S ribosomal subunit and initiation factors [53,54]. As functional IRESs are defined more reliably by activity rather than by primary sequence, bioinformatic prediction alone is insufficient; reporter assays, mutagenesis, ribosome profiling, and proteomic evidence remain important for validation [22,52,55].

This mechanism is particularly relevant to GBM because hypoxia, nutrient deprivation, oxidative stress, and anticancer therapy can suppress cap-dependent translation while favoring selective translation programs [56,57,58,59,60,61]. Several ITAFs are dysregulated in GBM, and oncogenic pathways such as EGFR, PI3K/AKT/mTOR, and RAS/MAPK can alter the abundance or activity of translation regulators [57,58,59,60,61]. Whether these pathways directly control specific circRNA IRESs remains incompletely defined, but the convergence of oncogenic signaling, stress, and cap-independent translation provides a plausible framework for selective circRNA protein production during tumor progression and treatment.

#### 3.2.2. m^6^A-Mediated Translation

CircRNA translation can also be initiated by N^6^-methyladenosine (m^6^A). m^6^A residues provide internal recruitment signals that can support cap-independent translation, with YTHDF3 and initiation factors including eIF4G2/DAP5 and eIF3A participating in ribosome recruitment [16,62,63,64]. m^6^A deposition and removal are dynamically regulated by writer and eraser proteins, and circRNA translation through this pathway can increase under cellular stress [16,65]. This is relevant in GBM, where METTL3, ALKBH5, FTO, and YTH-domain proteins have been linked to stem-cell maintenance, metabolic adaptation, invasion, and treatment response [66,67,68,69,70]. m^6^A modification also affects circRNA localization, stability, and RNA-protein interactions, indicating that epitranscriptomic regulation may coordinate several aspects of circRNA biology [63,71].

#### 3.2.3. Rolling Circle Translation

A feature unique to circular templates is rolling-circle translation. CircRNAs containing a continuous open reading frame without an in-frame stop codon can be traversed repeatedly by ribosomes, generating extended proteins composed of repeated peptide units [15,72,73]. Initiation generally still requires an internal recruitment mechanism such as an IRES or m^6^A, after which elongation can continue around the circular template. Endogenous examples remain less common than conventional circRNA translation, but the mechanism is biologically relevant in GBM because circEGFR has been reported to undergo rolling-circle translation to generate rtEGFR [25].

### 3.3. Experimental Approaches Used to Identify Translated CircRNAs

Establishing bona fide circRNA translation is technically demanding because most circRNAs arise from protein-coding genes and may share much of their sequence with linear mRNAs. Strong studies therefore combine orthogonal evidence demonstrating the circular transcript, ribosome engagement, production of a circRNA-specific protein product, and functional dependence on translation rather than on the RNA molecule alone [10,14,16,74]. No single assay is sufficient in every setting.

#### 3.3.1. Ribosome Profiling

Ribosome profiling (Ribo-seq) identifies ribosome-protected RNA fragments and can reveal footprints spanning the BSJ, which strongly supports engagement of a circular template [15,73]. However, ribosome occupancy does not by itself prove productive translation, and low circRNA abundance can limit BSJ-spanning reads. Ribo-seq is therefore best viewed as a discovery and supporting tool rather than definitive proof.

#### 3.3.2. Polysome Profiling

Polysome profiling provides complementary evidence by determining whether a circRNA associates with multiple translating ribosomes [75,76]. Redistribution after puromycin or initiation inhibitors can strengthen the interpretation, but polysome association alone remains insufficient because non-coding RNAs can associate with ribosomes without producing stable protein products.

#### 3.3.3. Mass Spectrometry

Mass spectrometry can provide highly specific evidence when peptides spanning a circRNA-derived junction are detected [14,16,74]. Customized protein databases containing predicted circRNA open reading frames improve discovery, although low protein abundance, rapid turnover, and a limited number of unique peptides can reduce sensitivity [74,77,78]. Consequently, a negative proteomic result does not necessarily exclude translation.

#### 3.3.4. Reporter Systems and Functional Validation

Reporter systems are useful for testing translational competence and dissecting IRES, m^6^A, or rolling-circle requirements [14,16,72,79,80]. Mutating start codons, IRES elements, m^6^A motifs, or continuous open reading frames can define mechanism, but artificial overexpression can create non-physiological translation. Reporter data should therefore be paired with endogenous validation.

#### 3.3.5. Genetic and Molecular Validation

Genetic approaches provide critical separation of RNA and protein functions. BSJ-directed siRNAs or ASOs can selectively deplete circular transcripts, while disruption of intronic sequences required for circularization can reduce circRNA formation without necessarily eliminating host-gene transcription [7,10,79,80,81]. Rescue with wild-type versus translation-deficient circRNAs is particularly informative because it tests whether the phenotype requires the encoded protein [14].

#### 3.3.6. Bioinformatic Prediction and Multi-Omics Integration

Computational resources such as circRNADb, riboCIRC, and TransCirc integrate open reading frames, IRES or m^6^A features, conservation, ribosome occupancy, and proteomic evidence to prioritize candidates [78,82,83,84]. Multi-omics integration can reduce false-positive assignments, but prediction cannot substitute for experimental confirmation. The most convincing designation of a translated circRNA therefore rests on convergent transcript-level, translation-level, protein-level, and functional evidence.

#### 3.3.7. Common Artifacts and Safeguards

Several technical artifacts can mimic circRNA formation or translation and therefore require orthogonal validation. RNase R resistance enriches for circular species but is not, by itself, proof of circularity because susceptibility varies with RNA structure and experimental conditions. Likewise, reverse-transcriptase template switching and rolling-circle cDNA synthesis can generate apparent BSJ-containing products or concatemers, making confirmation by independent approaches important [6,21,85]. BSJ detection by RNA-seq or Ribo-seq is also sensitive to alignment algorithms, mapping parameters, read length, and sequencing depth; consequently, BSJ-spanning ribosome footprints should be interpreted as evidence of ribosome engagement rather than definitive proof of productive translation [85]. For mass spectrometry, the strongest evidence is a peptide unique to the circRNA-encoded product, ideally spanning a junction or otherwise absent from proteins produced by linear splice isoforms or alternative proteoforms. Database composition and false-discovery thresholds should be specified, and candidate peptides should be validated where feasible using synthetic standards, circRNA-selective depletion, or complementary immunodetection [85]. Together, these considerations favor convergent evidence from transcript-level, ribosome-level, protein-level, and genetic approaches rather than reliance on any single assay.

#### 3.3.8. Contested and Unresolved Cases

Not all reported circRNA translation events have been independently confirmed, and several observations illustrate why candidate products should be interpreted cautiously. A prominent example is circZNF609, initially reported to associate with polysomes and produce a protein in a splicing-dependent, cap-independent manner. Subsequent analysis of commonly used circZNF609 overexpression constructs found that the detected protein products could persist when circRNA formation was disrupted and were most consistent with translation from linear or trans-spliced by-products; endogenous circZNF609-derived protein was not detected in that study [86]. More broadly, systematic testing of circRNA overexpression vectors has shown that cryptically spliced linear transcripts and concatemeric by-products can generate apparent translation products, including products that contain a back-splice-junction sequence and can therefore mimic rolling-circle translation [87]. Predicted open reading frames identified by sequence analysis, conservation, ribosome association, or isolated mass-spectrometric signals should therefore be regarded as candidates rather than established circRNA-encoded proteins unless circular origin and endogenous protein production are demonstrated independently. Apparent immunoreactivity also requires caution when antibodies can recognize shared sequences in canonical host-gene products or alternative proteoforms. These unresolved cases do not argue against circRNA translation as a biological phenomenon; rather, they underscore the need for orthogonal evidence, circRNA-selective genetic perturbation, translation-deficient rescue controls, and peptide or antibody reagents that distinguish the circular product from linear-transcript-derived proteins.

## 4. Protein-Encoding Circular RNAs in Glioblastoma

Only a small fraction of the thousands of circRNAs detected in GBM have been shown to encode functional proteins. Nevertheless, the best-supported examples already span receptor signaling, cell-cycle control, DNA repair, transcription, metabolism, stemness, and therapeutic response (Table 2; Figure 2). Their products can be either oncogenic or tumor suppressive, emphasizing that circRNA translation is not intrinsically pro-tumorigenic but instead expands the repertoire of regulatory proteins available to GBM cells.

### 4.1. circHGF

#### Discovery and Characterization

CircHGF is generated from the HGF locus and represents an oncogenic translated circRNA reported in GBM [23,88,89]. Expression studies showed increased circHGF in GBM relative to normal brain and an association with activation of the c-MET pathway. Saunders and colleagues demonstrated that circHGF encodes C-HGF, a novel HGF-related protein containing a unique junction-derived sequence that distinguishes it from the product of linear HGF mRNA [23]. Detection of this sequence supports translation from the circular template. Functionally, C-HGF stimulates c-MET signaling and downstream programs involved in GBM proliferation and survival. Silencing circHGF reduces growth, migration, and invasion, whereas increased circHGF promotes aggressive phenotypes; xenograft studies further supported a role in tumor growth [23]. The finding is notable because a circRNA-encoded product acts as a growth-factor-like extracellular signal rather than solely as an intracellular regulator. It therefore links non-canonical translation directly to a clinically relevant receptor tyrosine kinase pathway and suggests several possible points of intervention, including selective depletion of circHGF, inhibition of C-HGF, or blockade of c-MET signaling.

### 4.2. circFBXW7

CircFBXW7 was among the first translated circRNAs characterized in glioma and remains a prototypical tumor-suppressive example [18]. It encodes FBXW7-185aa, a 185-amino acid protein distinct from canonical FBXW7. The full-length FBXW7 pathway restrains several oncogenic proteins, and the circRNA-encoded product reinforces this tumor-suppressive network by antagonizing USP28-mediated stabilization of c-Myc [18]. Increased c-Myc degradation reduces expression of proliferative and metabolic programs required for tumor growth. CircFBXW7 is reduced in glioma specimens relative to non-neoplastic brain, while restoration of FBXW7-185aa suppresses proliferation and tumor growth in experimental models [18]. This study illustrates an important principle: a circRNA-encoded protein can influence a major cancer pathway through a mechanism distinct from, yet functionally complementary to, the canonical host-gene product.

### 4.3. circSHPRH

CircSHPRH encodes SHPRH-146aa, a 146-amino acid protein that supports the tumor-suppressive activity of full-length SHPRH [19]. SHPRH is an E3 ubiquitin ligase involved in genomic maintenance and DNA-damage responses. SHPRH-146aa protects the full-length protein from ubiquitin-mediated degradation, increasing its stability and thereby preserving tumor-suppressive function [19,90]. CircSHPRH expression is reduced in glioma specimens, and restoration of SHPRH-146aa inhibits tumor growth in vitro and in vivo [19]. The circSHPRH model is particularly relevant to treatment biology because genomic stability and DNA-repair capacity can influence sensitivity to radiation and alkylating agents. At the same time, the available data support a tumor-suppressive rather than a simple resistance-promoting role, illustrating why the biological effect of each translated circRNA must be evaluated individually. Mechanistically, SHPRH promotes K63-linked polyubiquitination of PCNA at stalled replication forks, supporting an error-free template-switching pathway of DNA-damage tolerance [90]. Thus, although SHPRH-146aa is tumor suppressive in the reported glioma models, stabilization of full-length SHPRH could also increase the capacity of tumor cells to tolerate particular forms of replication-blocking DNA damage. The net effect of circSHPRH/SHPRH-146aa on response to TMZ or radiation therefore cannot be predicted from its tumor-suppressive phenotype alone and requires direct experimental testing.

### 4.4. circAKT3

CircAKT3 produces AKT3-174aa, a tumor-suppressive protein with biological activity that contrasts with the conventional growth-promoting role of AKT signaling [20]. Translation was supported by back-splice-junction analysis, tagged expression constructs, and detection of circRNA-specific peptides. Mechanistically, AKT3-174aa interacts with phosphoinositide-dependent kinase-1 (PDK1) and interferes with phosphorylation required for maximal AKT activation, thereby restraining PI3K/AKT signaling [20]. Restoration of circAKT3 or AKT3-174aa reduces proliferation, clonogenic growth, migration, invasion, and orthotopic tumor growth, whereas depletion of circAKT3 increases AKT activity and malignant phenotypes. Clinical analyses further associated reduced circAKT3 abundance with higher-grade glioma and poorer outcome [20]. Because PI3K/AKT signaling is frequently activated in GBM through receptor tyrosine kinase alterations, PTEN loss, and other mechanisms, AKT3-174aa represents an endogenous brake on a central oncogenic pathway. It also illustrates how circularization can generate a protein whose function diverges substantially from the canonical product of the same genomic locus.

### 4.5. circLINC-PINT

CircLINC-PINT provides a different example because it arises from the LINC-PINT locus, previously characterized primarily as a non-coding RNA [91,92]. The circular transcript contains an open reading frame encoding the 87-amino acid peptide PINT87aa [21]. Translation was supported by circRNA-specific constructs, epitope tagging, mass-spectrometric peptide detection, and functional rescue experiments indicating that the biological activity depends on the encoded peptide. PINT87aa localizes to the nucleus and interacts with the RNA polymerase II-associated PAF1 complex, which regulates transcriptional elongation and chromatin-associated processes [21,93]. Through this interaction, PINT87aa suppresses elongation of oncogenic transcripts and restrains glioma growth. The finding expands the conceptual range of translated circRNAs by showing that they can produce nuclear regulatory peptides from loci not traditionally considered protein coding and can influence cancer through control of transcription rather than receptor or kinase signaling.

### 4.6. circNTRK2

CircNTRK2 encodes NTRK2-243aa, a tumor-suppressive protein whose function differs from that of the full-length TrkB receptor [24]. Rather than promoting neurotrophin signaling, NTRK2-243aa regulates tumor metabolism. NTRK2-243aa suppresses aerobic glycolysis by promoting PAX5 phosphorylation at Y102 and subsequent degradation, thereby reducing HK2 and PKM2 expression [24]. These metabolic changes are accompanied by decreased GBM proliferation, migration, and invasion, whereas circNTRK2 depletion produces the opposite phenotype. The study therefore connects circRNA translation to metabolic plasticity, an important feature of GBM cells growing in regions with fluctuating oxygen and nutrient availability. It also reinforces the principle that the function of a circRNA-encoded protein cannot be inferred simply from the canonical protein encoded by its host locus.

### 4.7. circEGFR

CircEGFR is distinctive because it undergoes rolling-circle translation to generate rtEGFR, a noncanonical EGFR-related protein [25,94,95,96]. A continuous open reading frame allows repeated traversal of the circular template, producing a product with sequences that distinguish it from full-length EGFR. Unlike circRNAs that encode a discrete protein of defined length, rolling-circle translation of circEGFR generates polymeric rtEGFR products containing repetitive EGFR-derived sequences rather than a single fixed-length protein. Translation and protein production were supported by circRNA-specific expression, ribosome association, peptide detection, and biochemical characterization [25]. rtEGFR promotes GBM proliferation, viability, motility, and tumor growth and sustains MAPK/ERK and PI3K/AKT signaling. Unlike canonical EGFR, whose activity is shaped by ligand availability, receptor internalization, and degradation, rtEGFR can sustain downstream signaling through a different regulatory context [25]. This has potential therapeutic significance because persistent signaling from a circRNA-derived EGFR product could reduce dependence on conventional receptor regulation and contribute to resistance to EGFR-directed strategies. CircEGFR therefore provides both a mechanistic example of endogenous rolling-circle translation in cancer and a direct connection between non-canonical translation and a dominant GBM oncogenic pathway.

### 4.8. circHEATR5B

CircHEATR5B encodes the relatively large HEATR5B-881aa protein and functions as a tumor suppressor in GBM [26]. Its size is notable because many early circRNA-encoded products were described as short microproteins, whereas HEATR5B-881aa illustrates that circular templates can encode much larger proteins. Translation was supported by circRNA-specific expression systems, ribosome association, and protein detection [26]. Restoration of circHEATR5B or HEATR5B-881aa inhibits proliferation, migration, clonogenic growth, and tumor formation, while circRNA depletion enhances malignant behavior. Mechanistically, HEATR5B-881aa suppresses glycolytic metabolism through the JMJD5/PKM2 axis [26,97,98,99,100]. PKM2 is a central regulator of glycolysis and also contributes to transcriptional programs that support cancer-cell adaptation. Together with NTRK2-243aa, HEATR5B-881aa indicates that translated circRNAs can shape metabolic state as well as classical signaling pathways, influencing how GBM cells adapt to the metabolically heterogeneous tumor microenvironment.

## 5. CircRNA-Derived Proteins as Regulators of GBM Biology

Accumulating evidence indicates that circRNA-encoded proteins regulate several core features of GBM biology (Figure 3). To avoid repeating the molecule-by-molecule descriptions in Section 4, this section integrates these findings by pathway node and biological consequence. A central theme is bidirectional regulation: translated circRNAs can either reinforce oncogenic networks or impose endogenous restraints on them, adding a layer of proteomic regulation to the genomic and transcriptional heterogeneity of GBM.

### 5.1. Receptor Tyrosine Kinase Input and Growth Signaling

C-HGF and rtEGFR act at the receptor tyrosine kinase input layer, reinforcing c-MET and EGFR signaling, respectively [23,25]. In contrast, AKT3-174aa restrains signaling downstream of receptor activation by interfering with PDK1-dependent AKT activation [20]. Viewed together, these proteins illustrate how translated circRNAs can modulate the same broad growth-signaling architecture at extracellular, receptor-associated, and intracellular nodes, contributing to heterogeneous pathway output and therapeutic response (Figure 3A).

### 5.2. Protein Stability and Transcriptional Control

Additional circRNA-encoded proteins act through protein stability and transcriptional control. FBXW7-185aa antagonizes USP28-dependent c-Myc stabilization, SHPRH-146aa stabilizes full-length SHPRH, and PINT87aa interacts with the PAF1 complex to restrain oncogenic transcriptional elongation [18,19,21]. These mechanisms connect circRNA translation to control of oncogenic protein abundance, genomic maintenance, and transcriptional output without requiring direct modulation of receptor signaling (Figure 3A,B).

The biological consequences of these pathway-level effects include altered proliferation, survival, and tumor growth. Importantly, the available examples include both oncogenic and tumor-suppressive products, indicating that the net contribution of circRNA translation depends on which translated circRNAs are expressed in a given cellular state rather than on translation from circular templates per se.

### 5.3. Metabolic Regulation

NTRK2-243aa and HEATR5B-881aa converge on glycolytic control through distinct mechanisms. NTRK2-243aa promotes PAX5 degradation and reduces HK2 and PKM2 expression, whereas HEATR5B-881aa suppresses the JMJD5/PKM2 axis [24,26]. Both reduce glycolytic activity and malignant phenotypes, linking circRNA translation to metabolic programs that support GBM growth and adaptation. Whether hypoxia, nutrient limitation, or therapy alters translation of these circRNAs remains an important unresolved question.

### 5.4. Regulation of Glioma Stem Cells

Glioma stem cells (GSCs) are implicated in tumor initiation, recurrence, and treatment resistance [101,102,103,104,105,106]. Direct studies of circRNA-encoded proteins in defined GSC states have not been systematically evaluated, but several validated proteins regulate pathways central to stem-cell maintenance. C-HGF, FBXW7-185aa, and NTRK2-243aa may influence stem-like phenotypes through c-MET signaling, c-Myc regulation, and metabolic control, respectively (Figure 3D) [18,23,24]. AKT3-174aa may also affect stem-cell-associated programs through its inhibitory effects on AKT signaling [20]. Determining whether specific circRNAs are selectively translated in therapy-persistent or stem-like states may clarify whether non-canonical translation contributes to cellular plasticity.

### 5.5. Regulation of Genomic Stability

CircSHPRH provides one of the clearest links between circRNA translation and genomic stability in GBM. Its encoded protein, SHPRH-146aa, protects full-length SHPRH from proteasomal degradation, thereby preserving the tumor-suppressive functions of the parental protein [19,90]. SHPRH participates in the cellular response to DNA damage, in part through regulation of proliferating cell nuclear antigen (PCNA) ubiquitination. Loss or reduced expression of circSHPRH and SHPRH-146aa could therefore compromise these protective mechanisms and favor the accumulation of genomic alterations that contribute to tumor evolution (Figure 3E). At present, however, comparable direct links between other GBM circRNA-encoded proteins and maintenance of genomic stability await further investigation.

### 5.6. Emerging Biological Functions of CircRNA-Derived Proteins

The currently characterized proteins likely represent only a small portion of the translated circRNA proteome. Their functions already include extracellular signaling, transcriptional regulation, control of protein stability, kinase modulation, and metabolic regulation, suggesting that additional biological activities will emerge as candidate proteins are validated. Functional studies should prioritize endogenous expression, physiological protein abundance, and separation of protein-dependent effects from non-coding actions of the same circular RNA. This distinction is essential because a circRNA may possess multiple functions that cannot be assigned from overexpression experiments alone.

## 6. CircRNA Translation and Therapeutic Resistance

Therapeutic resistance is the major barrier to durable control of GBM. Surgery, radiation, and TMZ impose strong selective pressures on a heterogeneous tumor in which stem-cell states, compensatory signaling, metabolic adaptation, DNA-damage responses, and translational reprogramming can support survival (Figure 4) [1,2,3,4,104,106,107,108,109,110,111]. CircRNA-derived proteins are particularly interesting in this context, as many are produced by cap-independent mechanisms that may remain active when conventional translation is abrogated [14,15,16]. This could allow selected proteins to be maintained under conditions that reduce global protein synthesis. Although mechanistic and pathway-level studies support a potential role for circRNA translation in treatment resistance, its broader contribution to therapeutic adaptation in GBM has yet to be fully established.

### 6.1. Resistance to Temozolomide

TMZ resistance is multifactorial and involves MGMT-mediated repair of alkylation damage, alterations in DNA-damage responses, persistence of stem-like tumor-cell populations, metabolic adaptation, and activation of prosurvival signaling pathways [112,113,114,115]. CircRNA-derived proteins could intersect with several of these processes and thereby influence the cellular response to TMZ. SHPRH-146aa stabilizes full-length SHPRH and preserves its tumor-suppressive functions, including regulation of PCNA ubiquitination [19]. In contrast, the oncogenic products C-HGF and rtEGFR reinforce c-MET- and EGFR-associated signaling, respectively, providing potential mechanisms through which translated circRNAs could promote survival and proliferative recovery following therapeutic stress [23,25].

Tumor-suppressive circRNA-encoded proteins may exert opposing effects. AKT3-174aa limits AKT activation, whereas FBXW7-185aa promotes c-Myc degradation, thereby restraining two signaling programs closely associated with GBM cell growth and survival [18,20]. NTRK2-243aa and HEATR5B-881aa suppress glycolytic pathways through distinct mechanisms involving PAX5 and JMJD5/PKM2, respectively [24,26]. Because metabolic flexibility can support tumor-cell survival under therapeutic stress, loss of these tumor-suppressive products could favor cellular states better able to tolerate TMZ exposure. Thus, the effect of circRNA translation on TMZ response is unlikely to be uniformly pro- or anti-resistant, but may instead depend on the balance of oncogenic and tumor-suppressive proteins expressed within a particular tumor or cell state. Although these mechanistic relationships provide a rationale for linking circRNA translation to TMZ response, a direct causal role for most of these proteins in TMZ resistance has yet to be established. This distinction is important because effects on proliferation, survival signaling, or metabolism do not necessarily translate into altered TMZ sensitivity.

### 6.2. Radiation Resistance

Radiation resistance is likewise shaped by efficient DNA-damage repair, GSC enrichment, stress signaling, and changes in the tumor microenvironment [106,116,117,118]. CircSHPRH is particularly relevant because SHPRH-146aa stabilizes a protein involved in genomic maintenance [19,90], although the direction of any effect on treatment response cannot be inferred from this function alone. Because SHPRH-dependent PCNA polyubiquitination promotes lesion bypass at stalled replication forks, preservation of SHPRH could, in some contexts, enhance tolerance of therapy-induced DNA damage; conversely, loss of this genome-protective pathway may increase damage sensitivity while also promoting genomic instability. Direct studies of TMZ- and radiation-treated GBM models are therefore needed to define the net effect of SHPRH-146aa on therapeutic response. C-HGF/c-MET signaling may also support post-radiation survival through pro-growth and stemness pathways [23]. At present, however, it is unclear whether irradiation selectively increases or decreases translation of these circular RNAs. Time-resolved studies of circRNA abundance, ribosome engagement, and protein output after radiation would help distinguish effects on circRNA biogenesis from regulation of translation itself.

### 6.3. Resistance to Targeted Therapies

Resistance to targeted therapy is common in GBM because inhibition of a single pathway often triggers compensatory signaling or selection of alternative cellular states [119,120,121,122]. C-HGF and rtEGFR provide plausible alternative inputs into c-MET or EGFR-associated networks [23,25]. rtEGFR is especially notable because persistent signaling from a noncanonical EGFR-related product could reduce dependence on conventional receptor regulation. At the same time, tumor-suppressive circRNA products modulate downstream AKT, c-Myc, transcriptional, and metabolic nodes, illustrating how the translated circRNA repertoire might influence pathway dependence and drug sensitivity. Determining whether specific circRNA-encoded proteins are enriched after targeted inhibition could reveal adaptive mechanisms that are invisible to DNA-level profiling.

### 6.4. Adaptive Translational Programs in GBM

The strongest conceptual link between circRNA translation and resistance is translational plasticity. Cellular stress, nutrient limitation, and mTOR pathway inhibition can suppress eIF4F-dependent protein synthesis while allowing selective translation through IRES- or m^6^A-dependent mechanisms [13,16,56,123,124,125,126,127,128,129]. Because many circRNAs use these alternative initiation routes, their encoded proteins may continue to be produced [129,130,131]. A well-defined precedent in GBM comes from linear mRNAs: mTOR inhibition activates hnRNP A1-dependent IRES-mediated translation of cyclin D1 and c-MYC, allowing continued synthesis of these cell-cycle regulators despite suppression of cap-dependent translation [132]. In the same adaptive pathway, m^6^A modification of the cyclin D1 and c-MYC IRESs enhances hnRNP A1 binding and contributes to resistance to mTOR inhibition [133]. Independent work provides an additional GBM-specific example: Blau and colleagues showed that c-Jun is translated through a 5′-UTR IRES in GBM cells, and c-Jun translation persisted when cap-dependent translation was suppressed by PI3K inhibition or rapamycin [134]. Although these examples involve linear rather than circular RNAs, they demonstrate that GBM cells can engage alternative initiation mechanisms during therapeutic stress and provide a mechanistic precedent for testing whether translated circRNAs are regulated similarly. This provides a mechanistic rationale for studying circRNA translation during therapy and is particularly relevant to GBM, where regional hypoxia and nutrient deprivation coexist with treatment-induced stress. The key unanswered questions are which circRNAs are preferentially translated under these conditions, whether their protein output is quantitatively sufficient to alter treatment response, and whether disrupting specific translation events produces therapeutic benefit in orthotopic and patient-derived models.

## 7. Clinical Implications

Translated circRNAs have several features that could support clinical applications, including transcript stability, context-dependent expression, and the generation of peptide sequences not found in proteins encoded by linear mRNAs [10,17,27,28,41,74,133,134]. These properties create opportunities for biomarker development, liquid biopsy, selective RNA targeting, direct protein inhibition, and immunotherapy (Table 3). The same characteristics that make circRNAs biologically interesting, however, also create analytical challenges: many products are low abundance, circular and linear transcripts share extensive sequences, and tissue-specific expression must be distinguished from tumor specificity. Most applications therefore remain preclinical and require validation in large, molecularly annotated patient cohorts.

### 7.1. Diagnostic and Prognostic Biomarkers

CircRNA expression patterns can differ between normal brain and GBM and may correlate with tumor grade, subtype, or outcome [27,41,135]. Several tumor-suppressive translated circRNAs are reduced in glioma, whereas oncogenic examples such as circHGF and circEGFR are increased in aggressive settings. The encoded proteins could provide an additional biomarker layer because junction-derived peptide sequences may be absent from the canonical proteome [74,77,129]. Antibodies or targeted mass-spectrometric assays recognizing these sequences could provide protein-level evidence of translation in clinical specimens. Such markers might complement established classifiers such as IDH status, MGMT promoter methylation, EGFR amplification, and TERT promoter mutation [46,136]. Clinical utility, however, will depend on assay reproducibility, sufficient abundance, and independent prognostic or predictive value beyond existing molecular features.

### 7.2. Liquid Biopsy Applications

The stability of circRNAs also makes them attractive for liquid biopsy. CircRNAs are detectable in extracellular vesicles, plasma, and cerebrospinal fluid, and GBM-derived extracellular vesicles can enter accessible biofluids [10,137,138,139]. Tumor-associated circRNAs could therefore provide minimally invasive markers of disease burden, treatment response, or recurrence. CircRNA-derived proteins may offer an additional level of specificity if junction-derived peptides can be detected reliably. Longitudinal measurement is particularly appealing because it could, in principle, capture dynamic changes during therapy. The major challenge is analytical sensitivity: many translated circRNA products are low abundance, and circulating signals must be distinguished from the high background of normal neural and systemic RNAs and proteins.

### 7.3. Therapeutic Targeting of Translated CircRNAs

The BSJ provides a natural sequence for transcript-selective intervention. Antisense oligonucleotides (ASOs), siRNAs, or RNA-targeting CRISPR-Cas13 approaches can in principle recognize the BSJ and deplete a circular transcript while sparing the corresponding linear mRNA [10,140]. This distinction is especially attractive when the host gene has essential physiological functions. For oncogenic circRNAs such as circHGF or circEGFR, selective RNA depletion could reduce production of the pathogenic protein without directly suppressing the canonical gene product. Since the circular transcript may also have non-coding functions, therapeutic effects would need to be assigned carefully. Major practical barriers include efficient delivery throughout infiltrative brain tumors, crossing or bypassing the blood–brain barrier, maintaining tumor selectivity, and limiting off-target effects.

### 7.4. CircRNA-Derived Proteins as Drug Targets

CircRNA-derived proteins may also be targeted directly. Junction-derived amino acid sequences can create molecular features absent from canonical proteins [14,15,16,74]. Extracellular products such as C-HGF may be comparatively accessible to antibodies, while intracellular products could be approached with small molecules, peptide inhibitors, or targeted protein degradation if suitable binding surfaces are identified [23,141]. Direct protein targeting would have the advantage of focusing on the functional output of translation, but feasibility will depend on protein abundance, structure, subcellular localization, and whether the product is required for tumor maintenance. For tumor-suppressive circRNA-encoded proteins, restoration, stabilization, or functional mimetics represent complementary strategies. These strategies are still largely conceptual and should be prioritized only after rigorous target validation.

### 7.5. CircRNA-Derived Neoantigens and Immunotherapy

Translation across BSJs can generate peptide sequences absent from the normal linear proteome, raising the possibility of circRNA-derived neoantigens [142,143,144,145,146,147]. Such peptides could expand the repertoire of tumor-specific targets for vaccines or other immunotherapies. However, immunological relevance cannot be inferred from translation alone: candidate peptides must be processed, presented by major histocompatibility complex molecules, and recognized by T cells in the tumor setting. Demonstrating natural presentation of circRNA-encoded peptides in GBM is therefore an important prerequisite for clinical development.

### 7.6. Challenges and Opportunities for Clinical Translation

Several barriers apply across these clinical applications. The field lacks standardized criteria for confirming endogenous circRNA translation, many encoded proteins are present at low abundance, and most reported molecules have not been validated across large independent cohorts [74,77]. Therapeutic approaches must additionally address delivery, tumor specificity, off-target effects, and intratumoral heterogeneity. Continued advances in RNA chemistry, delivery systems, proteomics, and genome editing may improve feasibility, but translated circRNAs should presently be regarded as promising experimental targets rather than established clinical biomarkers or therapies.

## 8. Future Directions and Outstanding Questions

The field has progressed from asking whether circRNAs can be translated to determining which translation events are biologically meaningful in human tumors. The most important next steps are therefore not simply to enlarge catalogs of predicted open reading frames but to establish endogenous protein production, define the mechanisms controlling translation in specific cellular states, and test functional relevance in physiologically appropriate GBM models. Rigorous validation using complementary approaches—including junction-specific proteomics and genetic strategies that distinguish circular from linear host transcripts—will be essential for separating genuine circRNA-encoded proteins from experimental artifacts.

An equally important question is when and where circRNA translation occurs within GBM. Hypoxia, nutrient deprivation, metabolic stress, and therapeutic exposure may alter cap-independent translation, but their effects on endogenous circRNA translation are not yet well defined. Single-cell, spatial, and proteogenomic approaches will help determine whether translated circRNAs are associated with particular tumor-cell states or microenvironmental niches. Ultimately, studies in patient-derived and orthotopic models will be needed to establish whether these proteins influence tumor heterogeneity, metabolic adaptation, or therapeutic response. The next phase of the field will therefore depend less on demonstrating that circRNA translation is possible and more on defining when it occurs, how it is regulated, and whether its products materially influence GBM biology.

The prevalence of circRNA translation in GBM remains unknown. Thousands of circRNAs are expressed, but only a small number have been assigned well-validated protein products [15,16,27,74]. Integrating long-read RNA sequencing with Ribo-seq, targeted proteomics, and BSJ computational pipelines should improve candidate discovery. However, the field must avoid equating predicted open reading frames or ribosome association with productive translation. Stringent orthogonal validation, ideally including detection of circRNA-specific peptides and genetic separation of circular and linear transcripts, will be necessary to define the true translated circRNA landscape.

A second priority is to distinguish functional drivers from incidental translation. The established proteins span oncogenic and tumor-suppressive activities, but it is unclear how many candidate products materially influence GBM fitness. Loss-of-function studies directed specifically at circular transcripts, combined with rescue using translation-competent and translation-deficient constructs, will be especially important for assigning protein-dependent functions. Endogenous tagging may further help establish localization and abundance. CircHGF illustrates the potential impact of this approach by showing that a circRNA can encode an extracellular activator of a major oncogenic receptor pathway [23].

The mechanisms controlling translation efficiency in GBM are also incompletely understood. IRES- and m^6^A-dependent initiation are established routes, yet the contribution of specific ITAFs, m^6^A regulators, oncogenic pathways, and microenvironmental stress remains to be defined for individual circRNAs [15,16,53,54]. Determining whether EGFR, c-MET, PI3K/AKT/mTOR, hypoxia, nutrient limitation, oxidative stress, or therapy selectively remodel the translated circRNA repertoire could reveal new links between signaling and translational adaptation. It will also be important to distinguish regulation of circRNA abundance from regulation of translation efficiency. A closely related question is whether circRNA translation causally contributes to therapeutic resistance. Stress-responsive IRES and m^6^A translation provide a plausible mechanism for maintaining selected proteins when cap-dependent synthesis is suppressed [13,16,128,148]. Future studies should therefore measure circRNA abundance, ribosome occupancy, and protein output dynamically before and after TMZ, radiation, and targeted therapy. Selective interruption of specific translation events in orthotopic and patient-derived models will be necessary to determine whether the mechanism is functionally important rather than simply correlated with cellular stress.

GSC biology is another important frontier. Several validated circRNA-encoded proteins regulate c-MET, AKT, c-Myc, and metabolic pathways implicated in stemness, but their expression and translation across GBM cell states are poorly defined [18,20,23,24]. Single-cell and spatial approaches coupled to protein-level validation may help determine whether specific translated circRNAs are enriched in GSCs, invasive-edge populations, hypoxic niches, or therapy-persistent states [149,150]. Such studies could also clarify whether circRNA translation contributes to transitions among cellular states rather than simply marking them. Therapeutic exploitation will require both target validation and delivery. BSJ-directed ASOs, siRNAs, and RNA-targeting CRISPR systems offer transcript selectivity, whereas unique peptide sequences may support antibody, small-molecule, degradation, or immunologic strategies [10,140,141,142,143,144,145,146,147]. The most attractive targets will be those with tumor-restricted expression, a demonstrable requirement for GBM maintenance, and a tractable molecular feature that can be targeted without disrupting the physiological host gene. For intracranial disease, delivery and intratumoral distribution will likely be decisive determinants of success.

Finally, translated circRNAs should be incorporated into broader models of GBM heterogeneity rather than studied in isolation. Combining circRNA profiling with genomics, epigenomics, proteomics, metabolomics, and spatial or single-cell analyses can identify cell-state-specific translational programs and reveal associations with treatment response [27,149]. Unlike static DNA alterations, circRNA translation can reflect dynamic post-transcriptional adaptation. Such integration could eventually add a functional layer to precision neuro-oncology, provided that candidate biomarkers or targets demonstrate reproducible value beyond existing molecular classifiers and can be measured reliably in patient material.

## 9. Conclusions

Translated circRNAs expand the functional output of the GBM transcriptome beyond conventional linear mRNAs. The best-supported examples produce both oncogenic and tumor-suppressive proteins that regulate receptor signaling, c-Myc and AKT activity, transcription, DNA repair, metabolism, and other features of malignant behavior [18,19,20,21,22,23,24,25,26]. Their diversity illustrates that circRNA translation is not a single pathway with a uniform consequence but a mechanism that can generate distinct regulatory proteins from otherwise familiar genomic loci.

Their reliance on cap-independent translation is particularly relevant to the stressed GBM microenvironment and raises the possibility that selected circRNA-encoded proteins persist during therapeutic challenge. At the same time, unique BSJs and junction-derived peptides create opportunities for selective detection and intervention. These possibilities are compelling, but direct evidence linking circRNA translation to treatment resistance and clinical outcome remains limited, and rigorous endogenous validation is essential.

The next phase of the field should therefore emphasize functional prioritization rather than catalog expansion. Defining which circRNAs are translated in patient tumors, how their translation is regulated across cell states and treatments, and which encoded proteins are required for tumor maintenance will determine whether this emerging biology can be translated into useful biomarkers or therapies. With improved multi-omics, proteomic, and genetic approaches, translated circRNAs may ultimately provide a new window into GBM heterogeneity and adaptive resistance.

## Figures and Tables

**Figure 1:**
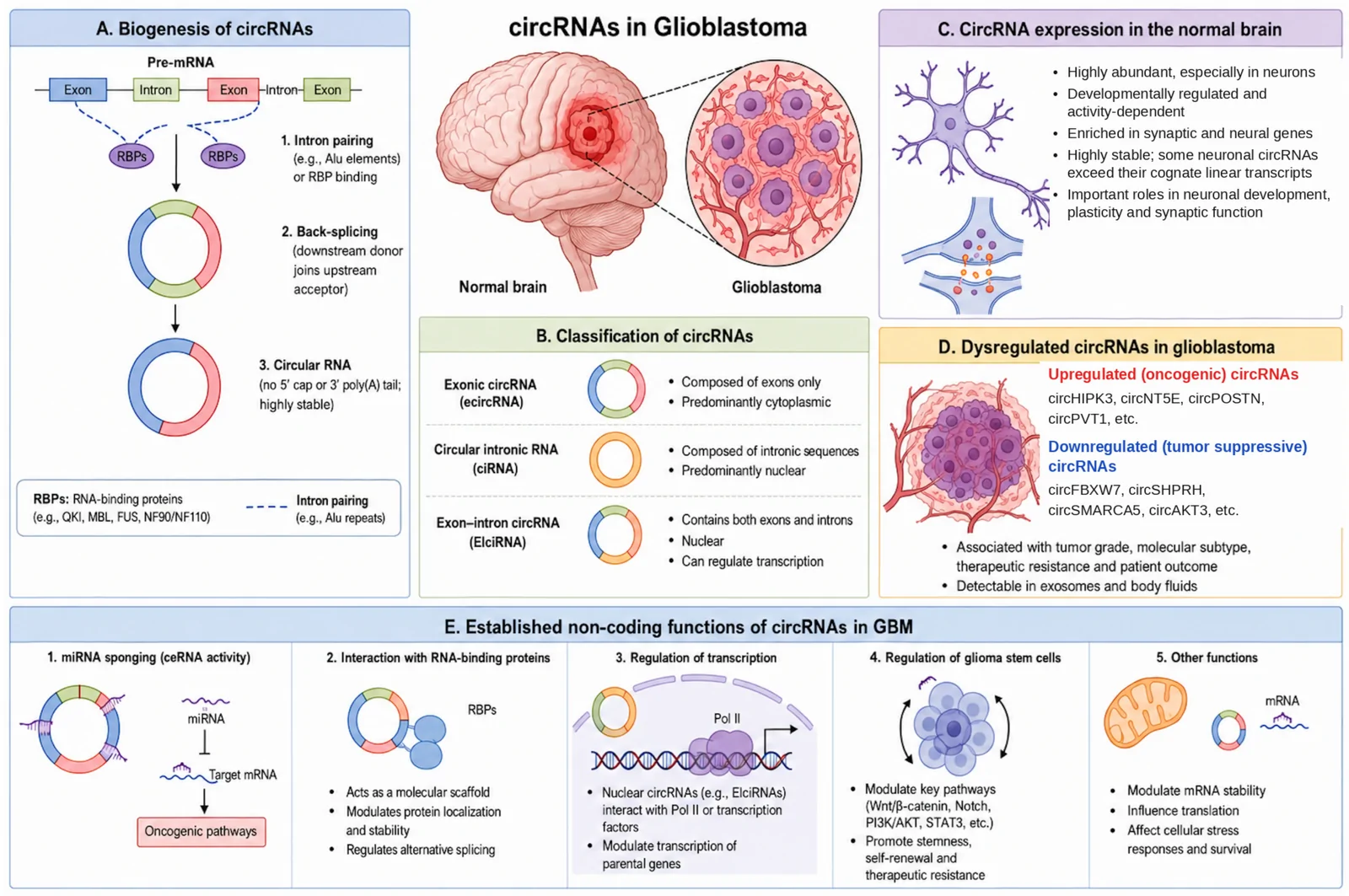
Circular RNAs (circRNAs) in glioblastoma: biogenesis, expression, classification, and established non-coding functions. (**A**) CircRNAs are generated by back-splicing, aided by complementary intronic sequences and RNA-binding proteins. (**B**) Major classes include exonic circRNAs, circular intronic RNAs, and exon-intron circRNAs. (**C**) CircRNAs are abundant in the normal brain, especially in neurons; many are developmentally regulated and highly stable, and some exceed their cognate linear transcripts. (**D**) GBM-associated circRNAs may be upregulated or downregulated and have potential biomarker value. (**E**) Established non-coding functions include microRNA sequestration (ceRNA, competitive endogenous RNA activity), RNA-binding protein interactions, transcriptional regulation, and modulation of stemness, RNA stability, translation, and stress responses.

**Figure 2:**
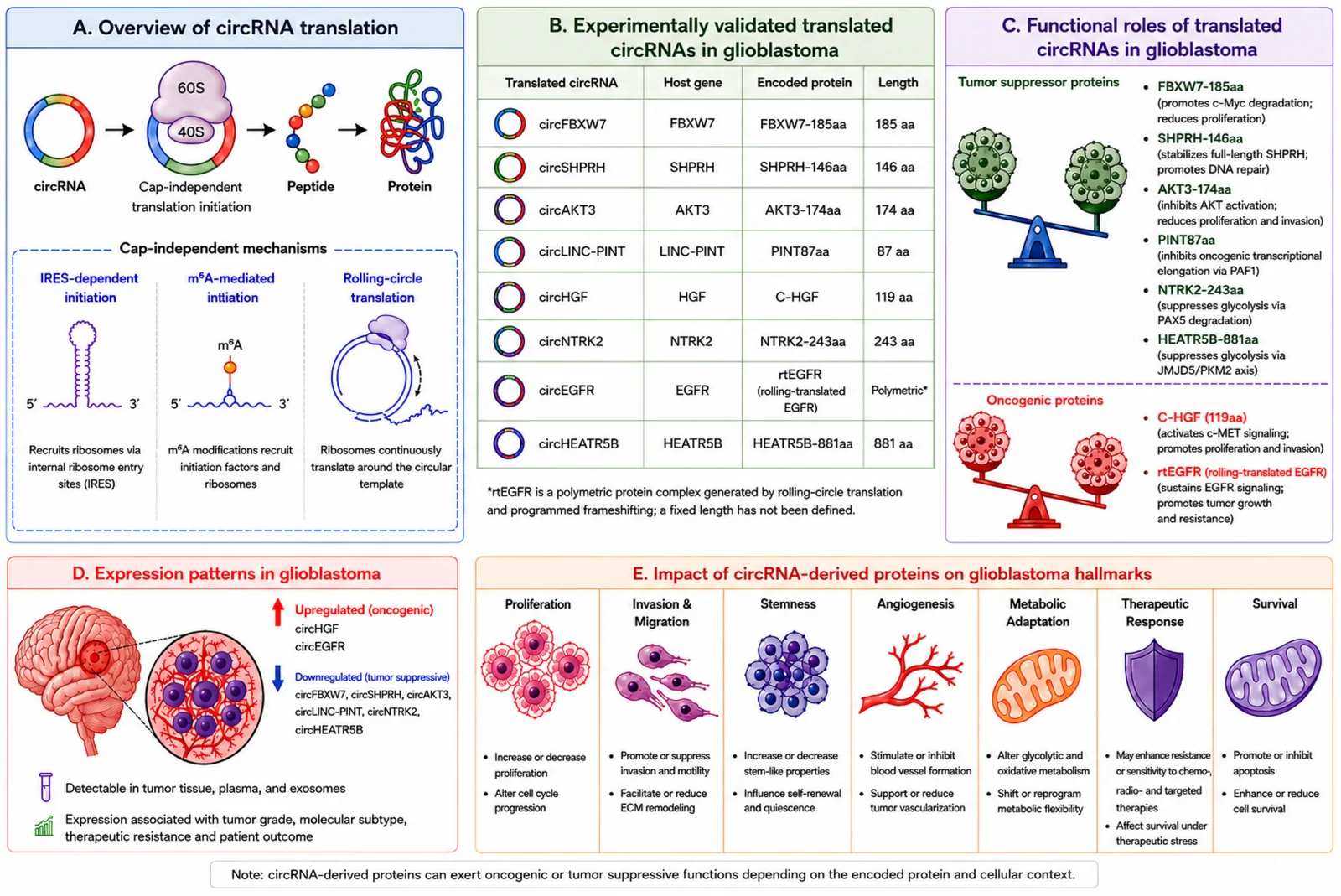
Overview of translated circRNAs in glioblastoma. (**A**) CircRNAs can undergo cap-independent translation through IRES-dependent, m^6^A-mediated, or rolling-circle mechanisms. (**B**) Reported glioma/GBM examples include circHGF/C-HGF, circFBXW7/FBXW7-185aa, circSHPRH/SHPRH-146aa, circAKT3/AKT3-174aa, circLINC-PINT/PINT87aa, circNTRK2/NTRK2-243aa, circEGFR/rtEGFR, and circHEATR5B/HEATR5B-881aa. (**C**) Their products include both tumor-suppressive and oncogenic proteins. (**D**) Representative expression patterns distinguish upregulated oncogenic circRNAs from downregulated tumor-suppressive circRNAs. (**E**) These proteins regulate signaling, proliferation, invasion, stemness, metabolism, and therapeutic response. Arrows indicate translational progression, regulatory relationships, or expression changes as labeled; the dashed circular arrow denotes repeated ribosome transit during rolling-circle translation, and colored circular structures represent the indicated circRNAs.

**Figure 3:**
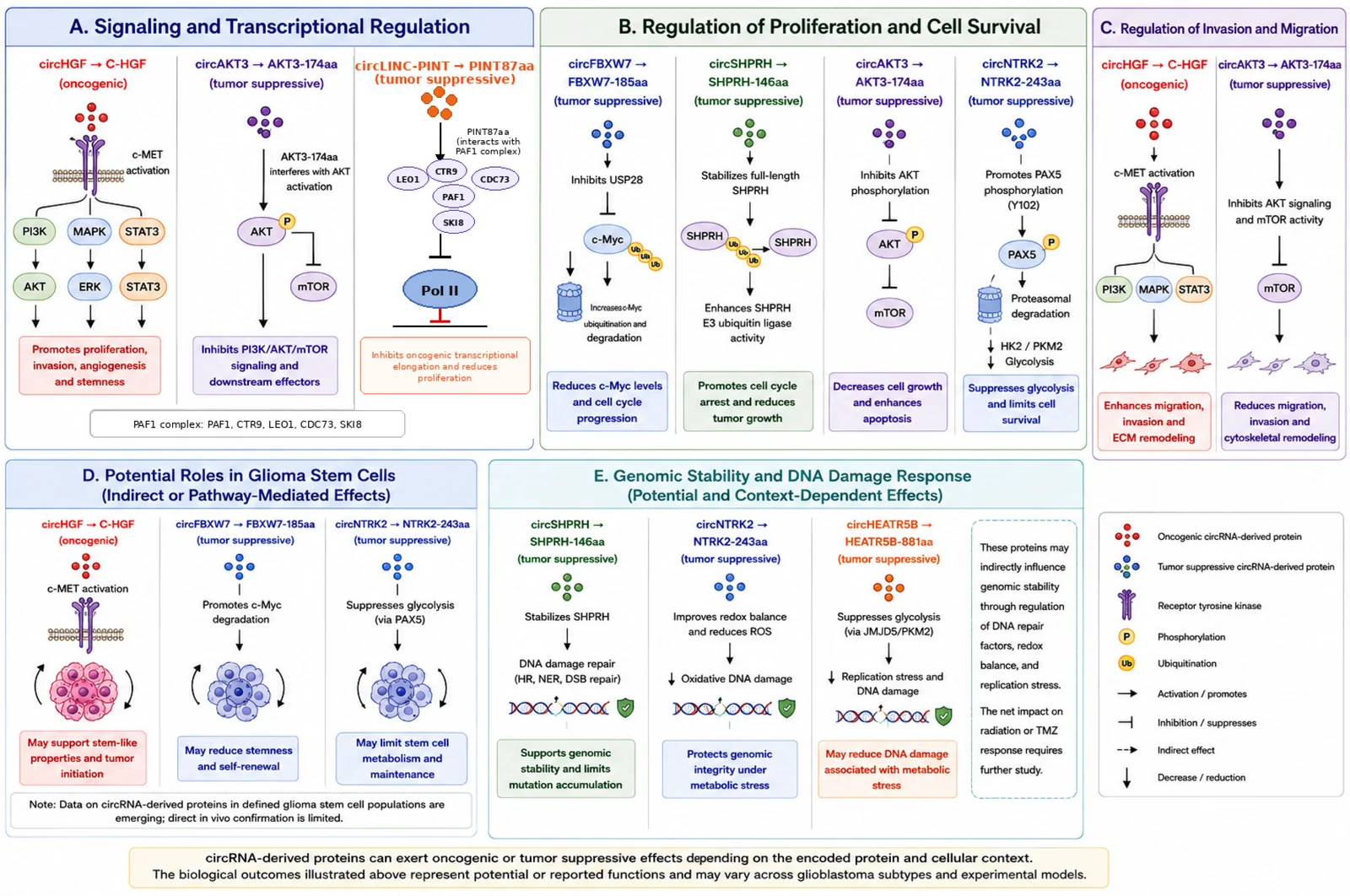
Molecular pathways regulated by circRNA-encoded proteins in glioblastoma. (**A**) Signaling and transcriptional regulation: C-HGF activates c-MET signaling, AKT3-174aa restrains PI3K/AKT signaling, and PINT87aa inhibits oncogenic transcriptional elongation through the PAF1 complex. (**B**) FBXW7-185aa, SHPRH-146aa, AKT3-174aa, and NTRK2-243aa suppress proliferation or survival through distinct mechanisms. (**C**) C-HGF promotes invasion and migration, whereas AKT3-174aa restrains these phenotypes. (**D**) Potential or indirect effects on glioma stem-cell biology may occur through c-MET, c-Myc, and metabolic pathways; direct GSC-specific evidence remains limited. (**E**) SHPRH-146aa is directly linked to genomic maintenance by stabilizing full-length SHPRH; effects of other circRNA-encoded proteins on genomic stability remain less well established. Graphical symbols and arrows indicate the molecular components and regulatory relationships defined in the symbol key within the figure.

**Figure 4:**
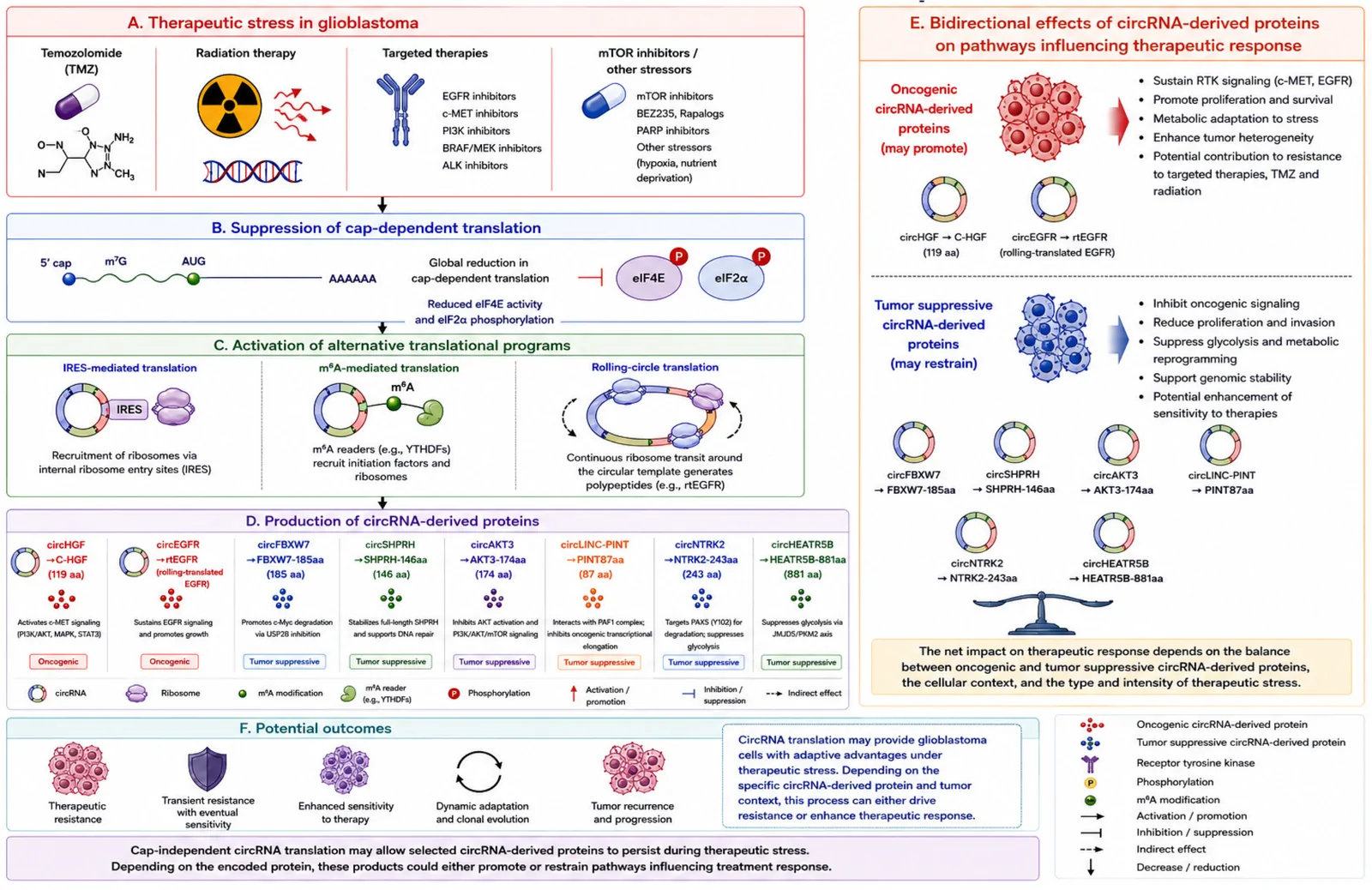
Potential contribution of circRNA translation to therapeutic resistance in GBM. (**A**,**B**) Therapeutic and microenvironmental stresses can suppress cap-dependent translation. (**C**) IRES-dependent, m^6^A-mediated, and rolling-circle mechanisms may permit selective circRNA translation during stress. (**D**) Representative products include oncogenic C-HGF and rtEGFR and tumor-suppressive FBXW7-185aa, SHPRH-146aa, AKT3-174aa, PINT87aa, NTRK2-243aa, and HEATR5B-881aa. (**E**) Their effects are bidirectional: oncogenic products may reinforce signaling and survival, whereas tumor-suppressive products may restrain growth, oncogenic signaling, or metabolic adaptation. (**F**) The net effect on treatment sensitivity, persistence, recurrence, or progression is therefore expected to depend on the translated-protein repertoire, cellular context, and therapeutic stress. The contribution of circRNA translation to GBM treatment resistance has yet to be fully defined. Graphical symbols, colors, and arrows indicate the molecular components, circRNA-derived protein classes, and regulatory relationships defined in the symbol key within the figure.

**Table 1:** Mechanisms and experimental approaches used to identify and validate translated circRNAs, with relevance to glioblastoma.

Category	Mechanism/Approach	Description	Key Evidence/Interpretation	Relevance to Glioblastoma
Translation Initiation Mechanisms	IRES-dependent translation	Internal ribosome entry sites recruit the translation machinery independently of the 5′ cap.	Functional IRES activity is supported by reporter assays together with mutational controls that disrupt the IRES while preserving circRNA production.	Provides a plausible route for circRNA-encoded protein synthesis when cap-dependent translation is reduced by hypoxia, nutrient limitation, or therapeutic stress.
	m^6^A-mediated translation	m^6^A modifications can recruit YTH-domain proteins and translation-initiation factors to promote cap-independent translation.	m^6^A-dependent circRNA translation has been demonstrated experimentally; site-directed mutation and perturbation of m^6^A readers/writers strengthen mechanistic assignment.	m^6^A machinery is dysregulated in GBM and may provide a stress-responsive route for circRNA translation; direct evidence for m^6^A-driven circRNA translation in GBM remains limited.
	Rolling-circle translation	CircRNAs with an open reading frame lacking an effective termination codon can support repeated ribosome transit around the circular template.	Continuous translation and multimeric products can be demonstrated using appropriately designed circular templates, junction-dependent assays, and protein analysis.	Provides a mechanism for amplified or repetitive protein production; rtEGFR is a GBM example of rolling-circle translation.
Methods for Identification of Translated circRNAs	Ribosome profiling (Ribo-seq)	Deep sequencing of ribosome-protected RNA fragments to identify ribosome-associated coding regions.	Back-splice-junction-spanning ribosome footprints support ribosome engagement of a circular transcript but do not by themselves prove productive protein synthesis.	Useful for genome-wide discovery and prioritization of candidate translated circRNAs in GBM; requires orthogonal validation.
	Polysome profiling	Fractionation of translating ribosome complexes, typically by sucrose-gradient centrifugation, followed by detection of circRNAs in ribosomal fractions.	Enrichment in heavy polysome fractions supports ribosome association and translational competence but is not definitive evidence of protein production.	Useful as an orthogonal method to support translational engagement of candidate GBM circRNAs.
	Mass spectrometry	Proteomic detection of peptides predicted from circRNA open reading frames.	Junction-spanning peptides that cannot arise from the cognate linear protein provide highly specific evidence of circRNA-encoded protein production.	Strong orthogonal evidence for endogenous circRNA translation, although sensitivity, peptide uniqueness, and database design can limit detection.
	Reporter assays	Engineered circRNA constructs coupled to epitope tags, fluorescent proteins, or luciferase reporters are used to test translation and initiation mechanisms.	Loss of reporter or protein production after mutation of the start codon, IRES, m^6^A site, or other required element supports mechanism; rigorous controls are needed to exclude linear-transcript artifacts.	Widely useful for mechanistic studies, but overexpression systems should be complemented by endogenous validation.
	Western blotting and immunodetection	Detection of endogenous or tagged proteins produced from candidate circRNAs.	Protein size and expression provide supportive evidence; circular origin is established most convincingly with junction-specific antibodies or genetic manipulations that distinguish circular from linear host-gene products.	Frequently used with reporter, proteomic, and genetic approaches to validate circRNA-encoded proteins in GBM.
Validation Strategies	Back-splice-junction mutagenesis	Disruption of sequences required for circularization or of the unique back-splice junction while minimizing effects on the linear host transcript.	Loss of the circRNA-encoded protein after selective disruption of circularization demonstrates dependence on the circular transcript.	Critical for distinguishing circRNA-encoded proteins from products of linear host-gene transcripts.
	Start-codon/ORF mutation	Mutation of the predicted initiation codon or coding sequence while preserving circRNA expression.	Loss of protein production or biological activity supports translation-dependent function rather than an RNA-only effect.	A strong validation strategy for separating coding from non-coding functions of GBM circRNAs.
	Loss- and gain-of-function studies	Selective manipulation of circRNA abundance and, where possible, rescue with coding-competent or translation-defective constructs.	Phenotypic rescue and separation-of-function experiments establish whether biological effects depend on the encoded protein.	Used to define the biological significance of translated circRNAs and to distinguish RNA-mediated from protein-mediated effects in GBM.

Abbreviations: circRNA, circular RNA; GBM, glioblastoma; IRES, internal ribosome entry site; m^6^A, N^6^-methyladenosine; ORF, open reading frame; Ribo-seq, ribosome profiling sequencing.

**Table 2:** Experimentally characterized translated circRNAs and their encoded proteins relevant to glioblastoma.

CircRNA (Host Gene)	Reported Expression in Glioma/GBM	Encoded Protein (Length)	Translation Mechanism	Key Evidence for Translation	Biological Function of Encoded Protein	**Major Signaling Pathways/Targets**	**Reference**
circHGF (HGF)	Upregulated	C-HGF (119 aa)	IRES-dependent	Cross-junction ORF identified by RNA-seq/ribosome profiling; functional IRES reporter analysis; circRNA-dependent C-HGF detection and secretion; protein output linked to c-MET activation.	Oncogenic; promotes GBM growth, motility, invasion, and intracranial tumor burden.	Activates c-MET and downstream PI3K/AKT, MAPK/ERK, and STAT3 signaling.	[23]
circFBXW7 (FBXW7)	Downregulated	FBXW7-185aa (185 aa)	IRES-dependent	Functional IRES reporter and mutational analyses; endogenous protein detected with specific antibody; LC-MS/MS identified a distinctive circRNA-encoded peptide.	Tumor suppressive; reduces proliferation, cell-cycle progression, and tumorigenicity.	Antagonizes USP28-mediated c-Myc stabilization, promoting c-Myc degradation.	[18]
circSHPRH (SHPRH)	Downregulated	SHPRH-146aa (146 aa)	IRES-dependent	IRES-driven ORF with overlapping coding sequence; circRNA-expression constructs and protein detection; endogenous SHPRH-146aa demonstrated in brain/GBM samples.	Tumor suppressive; stabilizes full-length SHPRH and reduces proliferation and tumorigenicity.	Preserves full-length SHPRH abundance, enabling SHPRH-dependent ubiquitination of PCNA.	[19]
circAKT3 (AKT3)	Downregulated	AKT3-174aa (174 aa)	IRES-dependent; overlapping start-stop codon	IRES reporter and IRES-mutant controls; ATG-mutant separation-of-function experiments; mass spectrometry confirmed AKT3-174aa peptides; junction-specific shRNAs reduced protein without altering linear AKT3.	Tumor suppressive; reduces proliferation, radiation resistance, and tumorigenicity.	Acts as a decoy for activated PDK1, thereby lowering phosphorylation of AKT at Thr308.	[20]
circLINC-PINT (LINC-PINT)	Downregulated	PINT87aa (87 aa)	IRES-dependent	RNC-seq identified translated circRNA candidates; circRNA-encoded peptide production validated experimentally; protein-dependent studies separated peptide from RNA effects.	Tumor suppressive; inhibits proliferation and tumor growth by restraining oncogenic transcription.	Interacts with the PAF1 complex and inhibits transcriptional elongation of oncogenic genes.	[21]
circNTRK2 (NTRK2)	Downregulated	NTRK2-243aa (243 aa)	IRES-dependent; overlapping start-stop codon	Functional IRES assays with mutant/truncated controls; circRNA-ORF expression and custom antibody detection; mass spectrometry confirmed NTRK2-243aa sequence.	Tumor suppressive; inhibits aerobic glycolysis, proliferation, and GBM progression.	Induces Y102 phosphorylation of PAX5, favoring its degradation and lowering HK2 and PKM2 expression.	[24]
circEGFR (EGFR)	Upregulated	rtEGFR (polymeric protein complex)	Rolling-circle translation; programmed -1 frameshifting	circEGFR confirmed by RNA-seq, Northern blot, and Sanger sequencing; protein products identified by antibody and LC-MS/MS; infinite ORF and rolling translation demonstrated biochemically.	Oncogenic; sustains EGFR signaling and tumorigenicity; depletion enhances response to EGFR-targeted therapy.	Binds EGFR, maintains membrane localization, and attenuates EGFR endocytosis/degradation.	[25]
circHEATR5B (HEATR5B)	Downregulated	HEATR5B-881aa (881 aa)	IRES-dependent	Functional IRES reporter, truncation, and mutation studies; circRNA-ORF constructs and custom antibody detection; mass spectrometry confirmed a unique HEATR5B-881aa peptide; start-codon/IRES perturbation separated protein from RNA effects.	Tumor suppressive; suppresses aerobic glycolysis, proliferation, and xenograft growth.	Promotes S361 phosphorylation and destabilization of JMJD5, thereby suppressing the JMJD5/PKM2 metabolic axis.	[26]

Abbreviations: GBM, glioblastoma; IRES, internal ribosome entry site; LC-MS/MS, liquid chromatography-tandem mass spectrometry; ORF, open reading frame; PAF1, polymerase-associated factor 1; PDK1, phosphoinositide-dependent kinase-1; RNC-seq, ribosome nascent-chain complex-bound RNA sequencing.

**Table 3:** Potential clinical applications of translated circRNAs and their encoded proteins in glioblastoma.

Clinical Opportunity	Biological/Molecular Rationale	Examples Relevant to GBM	Potential Application	Key Limitations/Requirements
A. Diagnostic and prognostic biomarkers	CircRNAs are stable, can be differentially expressed in GBM, and translated products may contain junction-derived peptide sequences absent from canonical proteins.	circHGF and circEGFR are oncogenic examples; circFBXW7, circSHPRH, circAKT3, circLINC-PINT, circNTRK2, and circHEATR5B are tumor-suppressive examples.	Use circRNA abundance and, where feasible, circRNA-encoded proteins or junction-specific peptides as complementary diagnostic or prognostic markers.	Require reproducible detection in patient specimens, independent validation in large molecularly annotated cohorts, and demonstration of added value beyond established GBM classifiers.
B. Liquid biopsy	CircRNAs are stable and can be detected in extracellular vesicles and biofluids. Secreted or released circRNA-encoded proteins may also be detectable.	GBM-associated circRNAs in plasma, extracellular vesicles, or cerebrospinal fluid; C-HGF is a secreted circRNA-encoded protein and illustrates the potential for extracellular detection.	Potential minimally invasive monitoring of disease burden, treatment response, or recurrence.	Analytical sensitivity and tumor specificity are major challenges. Detection of circRNA-encoded proteins in blood or CSF remains to be established for GBM.
C. Transcript-selective RNA targeting	The back-splice junction provides a sequence feature that can distinguish a circular transcript from its cognate linear mRNA.	BSJ-directed ASOs, siRNAs, or RNA-targeting CRISPR-Cas13 approaches directed against oncogenic circRNAs such as circHGF or circEGFR.	Selective depletion of pathogenic circRNAs while sparing the corresponding linear host-gene transcript; potential enhancement of therapeutic sensitivity.	Efficient delivery throughout infiltrative intracranial tumors, blood-brain-barrier constraints, off-target effects, and separation of RNA-mediated from protein-mediated functions.
D. Direct targeting or replacement of circRNA-encoded proteins	CircRNA-derived proteins may contain unique structural or junction-derived features not present in canonical host-gene products.	Inhibit oncogenic products such as C-HGF or rtEGFR; restore, mimic, or stabilize tumor-suppressive products such as FBXW7-185aa, AKT3-174aa, NTRK2-243aa, or HEATR5B-881aa.	Antibodies or other inhibitors for accessible oncogenic products; small molecules, peptides, targeted degradation, or replacement/mimetic strategies depending on the target.	Feasibility depends on protein abundance, structure, localization, druggability, and demonstration that the protein is required for tumor maintenance.
E. CircRNA-derived neoantigens and immunotherapy	Translation across back-splice junctions can generate peptide sequences absent from the normal linear proteome.	Potential junction-derived neoepitopes from translated circRNAs in GBM; natural presentation has not yet been established for the principal GBM examples reviewed here.	Possible future vaccine or T-cell-based targeting of naturally presented circRNA-encoded peptides.	Translation alone does not establish immunogenicity. Candidate peptides must be processed, presented by MHC molecules, and recognized by T cells in the tumor setting.
F. Precision neuro-oncology and patient stratification	CircRNA translation may provide a dynamic post-transcriptional layer of tumor heterogeneity that is not captured by DNA-level profiling alone.	Integration of circRNA profiling with genomics, epigenomics, proteomics, metabolomics, and spatial or single-cell analyses.	Potential identification of cell-state-specific translational programs, pathway dependence, and treatment-associated changes.	Clinical use will require standardized assays, robust patient-level reproducibility, and evidence that translated-circRNA measurements improve prediction beyond established molecular markers.

Abbreviations: ASO, antisense oligonucleotide; BSJ, back-splice junction; CSF, cerebrospinal fluid; GBM, glioblastoma; MHC, major histocompatibility complex.

## Data Availability

No new data were created or analyzed in this study. Data sharing is not applicable to this article.
